# “We are struggling to seek justice”: a study of the criminal justice system and transgender experiences in Pakistan

**DOI:** 10.1080/26895269.2024.2303478

**Published:** 2024-01-22

**Authors:** Alamgir Alamgir

**Affiliations:** School of Education, RMIT University, Melbourne, Australia

**Keywords:** Cheela, criminal justice system, guru, Hijra, Khawaja Sara, Pakistan

## Abstract

**Background:**

This article aims to explore the complex intersection of transgender people’s identities within Pakistan’s criminal justice system; a nation in South Asia with a vast population of around 220 million.

**Aim:**

This paper aims to explore the myriad challenges faced by transgender individuals when they navigate the realms of justice, encompassing encounters with community people, law enforcement, experiences within the prison system, interactions with prosecution, and engagements with the judicial process in Pakistan.

**Method:**

Employing a qualitative research methodology, this study draws on one set of semi structured interviews with ten members of the Khawaja Sara communities, representing transgender individuals, within their residential spaces in Peshawar.

**Findings:**

Through the lens of an intersectional framework, the study’s findings demonstrate the fragile nature of the criminal justice system in Pakistan as it fails to provide justice to transgender people and to safeguard their lives in Peshawar.

**Conclusion:**

The study demonstrates entrenched issues like transphobia, cisgenderism, and cisnormativity in the criminal justice system of Pakistan, contributing to suboptimal case handling and the exacerbation of hate crimes against the Khawaja Sara communities in Peshawar. Consequently, a considerable number of cases involving transgender individuals either go unreported or witness the voluntary withdrawal of First Information Reports (FIRs), perpetuating a cycle of impunity and significantly compromising the pursuit of justice in Peshawar, Pakistan.

## Introduction

In recent years in the field of criminology, gender studies are on its horizons, embracing a inclusive perspective and acknowledging the intersections of different social identities and their impact within the criminal justice system. One such intersectional lens increasingly given space is the exploration of transgender people identities, here in the socio-cultural context of postcolonial Islamic Pakistan. The present study discusses the multifaced realm of intersectional criminology, focusing on the Khawaja Sara and Hijra communities in Pakistan and their complex relationships with the criminal justice system.

Khawaja Sara and Hijra exhibit diverse and contested gender identities due to their heterogeneous nature. Some are assigned male at birth but live with a female identity, while others are born with diverse genitalia and identify as non-binary or intersex. Those assigned male may align with masculine behavior yet identify as Khawaja Sara or Hijra through feminine clothing and makeup (Awan, 2019). Nanda (1986) reported that Hijra’s have assigned male identity at birth, but prefer to live as women and often undergo gender affirming surgeries. They are culturally defined as ‘neither men nor women’ (Nanda, 1990), while Reddy (2005) described Hijra as ‘men who wear women’s clothes’ and are often perceived as homosexual. For Jami (2005), Hijra is an umbrella term that is interchangeably used for transgender people, intersex, bisexual, or gay individuals. Similar, Khan (2016) in his research work described that during the Mughal era, Khawaja Sara were the individuals who usually dressed and were addressed as men while Hijra were the individuals who dressed in feminine attire and performed dances and sang in the Mughal Harems. In this paper, I work with the proposition that Khawaja Sara and Hijra are individuals who are assigned male at birth, but they identify themselves as women in Peshawar Pakistan.

Transgender people’s identities are often associated with oppressed and marginalized communities that garner significant attention in the current academic literature. Global research scholarship has extensively explored the precarity of trans people’s lives, reporting that they are more prone to heightened levels of threat, vulnerability, and anxiety compared to their heterosexual cisgender counterparts (Benson, 2020; Miles-Johnson, 2016). In the UK, a study found that trans people are more likely to be unsupported by family, friends, and society (Walters et al., 2020). Other studies have demonstrated that transgender people in UK are more likely experience verbal harassment, physical violence, sexual abuse and assault comparing to the other general population (e.g., Ellis et al., 2016).

Likewise, research conducted in the USA and Australia, encompassing both qualitative and quantitative studies, have revealed that transgender individuals encounter significant challenges. These challenges encompass sexual assault, discrimination, stigma, harassment, mistreatment, and even a lack of justice within the criminal justice system (Badgett et al., 2007; Mogul-Adlin, 2015). Also, trans people’s lack of access to gender-affirming, sexual health/STI and mental health services, discrimination in housing and employment opportunities are the commonplace for many transgender people (Stotzer, 2009). Transgender people in African countries are not granted their due rights and they are largely treated as strangers on their own land (M’baye, 2013). While, in Türkiye transgender people are regularly targeted, tortured and ill-treated by the police forces. However, the strategies of the police to control trans women’s existence in the urban areas in Türkiye has moved away from inflicting direct physical violence, and toward employing ‘law’ and creating an omnipresence of law in their everyday lives (Taşcıoğlu, 2023).

In South Asia, transgender people are discriminated based on their gender and sexual identity. Their legal recognition as ‘third sex’ had been accepted after a long struggle and wait in countries like India, Pakistan, Nepal, and Bangladesh (Hossain, 2018). However, the rights of these people are being violated in their daily routine life despite their identities being recognized by different governments and protected by law. Some South Asian countries have already started to let trans people b involved in different social and economic activities. In 2013, transgender people were recognized as a third gender in Bangladesh (Islam, 2019). This recognition was aimed to protect the human rights of the third gender, enabling them to identify their gender as ‘Hijra’ in all government documents and passport. Section 27 of the Constitution of Bangladesh states that the citizen is equal before the law and are entitled to equal protection of law. But the legal protection of the human right of the newly recognized third gender is questionable till now. The Prevention of Oppression against Women and Children Act, 2000,^1^ describes the right of only (cisgender) women and children. Despite the recognition of transgender people in Bangladesh, they are becoming omnipresent victims of rape. However, unlike women and children, their rape cases are rarely filed, as police officers often disregard their gender diversity and fail to assign proper value to their experiences. This social taboo and negligence is costing transgender people their due rights like legal protection. Therefore, it is important to address this issue to create social awareness which might induce equal laws for every gender identity. In India, the prosecution of transgender people by the state and discrimination by society continued undebated. The law does not guard against police violence, although cases of physical and sexual violence by the police against the transgender people have been well-documented (Anuvinda & Siva, 2016; Mitra, 2018).

For, Khawaja Sara, Hijra living as trans is a difficult task in Pakistan. They are frequently marginalized in their daily life with less opportunities of education, health, and employment avenues (Saddique et al., 2017). Fearing physical attacks, when they run away from home at an early age to find acceptance and work in their closed group and community (de Lind van Wijngaarden et al., 2013). Hijra are discriminated against, which make them more precarious and vulnerable (Kilbride, 2015).

Therefore, Khawaja Sara and Hijra live challenging lives, resorting to begging, dancing, singing, and providing sex services for survival, further intensifying their vulnerability (Khan et al., 2008). They endure frequent abuse and harassment from individuals who visit their homes and engage their services for dancing, singing, or sex work. These individuals encompass their intimate partners, friends from schools or colleges during their youth, as well as relatives, neighbors, and teachers within their communities. The Human rights commission of Pakistan has reported that more than 500 of transgender women have faced physical and sexual violence, kidnapping, and have been murdered since 2015 in Peshawar city (HRCP, 2015).

The Express Tribune, a local newspaper, has reported on the assassination of a renowned transgender women while her friend sustained injuries during a firing assault in District Mardan, a region near to Peshawar city (Khan, 2022). Similarly, another newspaper reported on the attack of four transgender women who were shot and injured in Peshawar city (Kakakhel, 2022). Aurat Foundation^2^ a local nonprofit organization that works for the empowerment of women and transgender people, has reported that currently transgender people are faced up with different problems. They experience social exclusion and are discriminated in their daily life that resultingly affect them at public places like hospitals, education facilities and by the police forces (Majeedullah, 2016; HRCP. 2011). Gallup and Gilani’s (2010) study indicates that merely 14% of the Pakistani population express interest in forming relationships with transgender individuals. These relationships include making friendship, companionship, and to meet and greet transgender people in markets, mosques, and other places of social gatherings. This pervasive social norm and the corresponding community responses have marginalized trans people, with both law enforcement and the judiciary often not treating their cases seriously. This fragile response toward transgender person issues questions the criminal justice in Pakistan.

This article initially explores the vulnerable situations faced by transgender people in Peshawar when they are living within their parents and families, and the frequent violence they experience when they move away to their guru-cheela houses. It then delves into the response of police and other law enforcement institutions, highlighting the scarcity of information and empirical research regarding the lives of transgender individuals within the criminal justice in Pakistan^3^. This article offers empirical insights into the intersection of transgender people lives and the criminal justice system in Pakistan. It examines the vulnerabilities and fragility within the system that hinder the well-being and prosperity of transgender individuals. This contribution to queer criminology explores the characteristics of transgender individuals and their heightened risk of victimization based on sexuality and gender identity in the Pakistani context.

My discussion is grounded in the socio-cultural, religious, and legal landscape of Pakistan. It illustrates how the lives and identities of transgender individuals intersect with the criminal justice system in the country. The crimes associated with transgender people’s lives (i.e., sex working, dancing, and singing) are perpetuated through systemic cisgenderist values that are influenced by social, cultural, and religious factors. Employing an intersectional approach, this analysis aims to scrutinize the discrimination and oppression experienced by transgender individuals, as well as the biases they encounter in the handling of their cases within the criminal justice system in Pakistan.

Kimberlé Crenshaw’s theory of intersectionality (Crenshaw, 1989), introduced in the late nineties, provides a foundational framework for understanding how interconnected social categories, such as race, gender, and class, intersect and mutually reinforce each other to shape an individual’s experiences. In the context of this paper discussion of criminal justice and trans identities in Pakistan would help to unravel the complex web of systemic inequalities, shedding light on how multiple dimensions of identity converge to influence the unique challenges faced by transgender individuals, within the justice system in Peshawar city. Finally, intersectionality of trans identities is discussed within the domain of judiciary and district courts that further navigate the insecurity of transgender person lives in the legal proceedings in Pakistan, despite of recent legislation passed by the Government of Pakistan that insures equal rights to transgender people in Pakistan.

This study will contribute not only to academic discourse but also can inform policy and advocacy efforts aimed at fostering a more just and equitable criminal justice system for all, regardless of gender identity. Through a nuanced understanding of the challenges faced by Khwaja Sara and Hijra individuals, it will be possible to move toward a society where justice is inclusiver, and the criminal justice system becomes an instrument of empowerment rather than marginalization. In this way, trans people should enjoy the same rights and opportunities as cisgender individuals without facing discrimination based on their gender identity.

## Materials and methods

This article brings attention to the everyday marginalization and oppression of transgender people in Peshawar city, and further discuss the role of community, police, courts, and judiciary that exacerbate their problems. I argue that transgender individuals, being particularly vulnerable, lack adequate support in police stations and judiciary courts. This lack of support creates opportunities for offenders to harm trans people in their lives, including injuries, attempted murder, and even killings of transgender people in Peshawar.

To research this, a qualitative research methodology was proposed that is approved the by human research and ethics committee of Royal Melbourne Institute of Technology Australia.

For data collection 05 gurus and 05 cheelas were selected through snowballing sampling from the members of transgender people in Peshawar. Gurus are the senior members and cheelas are the young members living in a place known as guru-cheela houses in Peshawar. This guru-cheela relationship is assumed as of a mother and daughter relationship where the senior member as guru is responsible for security and providing space of living, and the cheela role is to earn through dancing and sex work to meet the expenses of the house. The qualitative research methodology was best suited for this study because it focused on engaging with the participants directly and allows for a meaningful analysis of their emotions and experiences (Cresswell, 2017). Additionally, snow balling technique was very effective to select the participants because they were hard to access, and they are also reluctant to mix with cisgender individual easily. Therefore, participants were referred by the senior members of the Khawaja Sara and Hijra communities in Peshawar. Following is the demographic profile of the participants who participated during the data collection process (Table 1).

**Table 1.** Demographic characteristics of participants.

Respondents	Age	Education	Status	Position
Saima	42	8^th^ Grade	Guru	Supervisor
Jameela	38	Illiterate	Guru	Supervisor
Sweetie	40	Bachelor of Arts (BA)	Guru	Supervisor
Kinza	41	5^th^ Grade	Guru	Supervisor
Shanza	25	Secondary School Certificate (SSC)	Cheela	Dancer
Kanwal	27	Illiterate	Cheela	Dancer/Singer
Rubi	20	Illiterate	Cheela	Dancer
Sheeza	22	6^th^ Grade	Cheela	Dancer/Actor
Nimo	30	Bachelor of Science (BSc)	Cheela	Dancer
Nirma	24	Secondary School Certificate (SSC)	Cheela	Dancer/Singer

The process of data collection was scheduled and completed in the months between May-September 2020. Before the interview session, participants were briefed on the aims and objectives of the study and their voluntary participation in the project. Initially, participants were contacted through phone calls and upon their agreement, face to face interview sessions were organized. All the interviews were recorded through audio recorder upon the consent provided by the participants. On completion of the face to face in depth interviews, all the interviews were translated and transcribed carefully from Pashto to English version by the researcher. During the data analysis, I used Pashto version of quotes to provide respect to participants wordings and to authenticate their responses in the data collection process. Additionally, direct Pashtu quotes is used to provide respect to the local cultural settings in Peshawar. Every participant is given a pseudonyms to ensure their security and protect their identity.

The data were thematic analyzed to examine and interpret the patterns and meanings in it. Braun and Clarke (2006) described that thematic analysis is a person-centred activity because researcher often divide the data into themes and sub-themes that can become the basis/unit of analysis. Doing this way, it developed a nuanced exploration of themes and patterns from the data, providing a rich and in-depth understanding of the research objectives. The analysis involved a recursive process, where data was initially coded into descriptive categories and subsequently refined through the identification and grouping of overarching themes. Rigorous attention was paid to maintaining the authenticity and context of the data, ensuring that the final themes accurately reflected the participants’ perspectives and experiences. Through this thematic analysis, the study aimed to uncover key insights, relationships, and recurrent motifs within the data, contributing to a comprehensive and nuanced interpretation of the research findings. The three themes, Intersection of trans identity with community, Intersection of trans identity with police system, and Intersection of trans identity with Judiciary and courts are discussed in the following sections.

## Results

### Intersection of trans identity with community

This section discusses transgender people’s lives within the domain of wider communities where they are living. The intersection of transgender people with community in Pakistan involves recognizing the complex socio-cultural, and religious factors that shape their experience in Pakistan. This includes their personal space that reflect transgender people’s relationships with their family members, close relatives, and peer groups, and their private space which reflects their relationships with the members of Khawaja Sara communities and their likeminded people. Nisar (2016) demonstrates that transgender people known as Khawaja Sara and Hijra are marginalized and discriminated within their parental houses for being trans in Pakistan. They are pressurized and even punished by their parents, family members and other close relatives to stop being trans and start living like cisgender people.

This was attested by Saima, who is a young cheela^4^ and explains how she escaped from her parent’s house:

My parents were not in favour of my trans identities. They beat me many times with sticks on my feet’s and back. “Za hapal plar da da koora hum obasali wama aow so shapy mi bahar hum kary v” (My parents discovered my trans identities, and they pushed me out of the house. I slept for several nights on the roads). They were very harsh to me when they discovered my trans identity.

Saima described the enormous pressure she faced from her family members because they were concerned that she was trans. As a result she left her family, and she was forced to leave her parental houses. This brought many new problems like life threatening situation for her. She stayed and passed several nights on roads because nobody was around to support her. I found that families remain rigid and behave harsh toward young transgender person because they consider them stigmata for their families when living in the strict socio-patriarchal cultural environment in Peshawar. This is the reason every transgender person interview gave for leaving their families: being pressurized and warned many times for being trans , they are physically beaten, and finally forced out to leave their parental house to avoid the stigma and ridicule.

Like Saima, the circumstances of Rubi are found similar where she shared her stories of pain, torture, and physically abuse in her family:

I was locked by my parents in a dark room for several days without food and water because of my trans behaviour. Daily I was brutally punished with sticks, rods, and belts. My parents wanted to take a pledge from me to not perform my trans behaviour and do not meet with the members of Khawaja Sara and Hijra communities. My parents warned all members of my family to do not provide any support in any capacity. My parental house was like a prison for me where I was detained/locked in the dark room for many days, but I did not leave my trans behaviour and was fighting till the last moment of my stay in my parental house.

Rubi illustrated the painful stories of her life that she faced during her stay within her parental house where she was targeted many times for being trans. According to Rubi, she was starved and put without food and water for several days as punishment. Similarly, Shanza shared the painful story of her deceased friend when she was shot dead by her boyfriend in a busy market in Peshawar city,

My friend, who was known for her innocence, went to her boyfriend to collect the money he had borrowed from her a few months earlier. Shockingly, he refused to repay the debt, and as my friend persisted, steadfast in her determination to receive what was owed, her boyfriend resorted to violence. Tragically, he shot her in the head and chest.

This painful story demonstrates the marginalized positionality of transgender people and the way cisgender people in the wider communities consider them an easy prey to target and seek to get rid of them. It is further alarming that violence against transgender people is not considered a serious crime, but is considered a routine day incident. Except few of the civil society organizations that are working for the right of transgender people in Peshawar, not many human rights organizations consider them as part of their developmental scheme. Therefore, only members of transgender communities sometimes come forward and record their protest in a press or media conference, but they do not get any attention in the media or in general public. This shows the extreme nature of community toward transgender people in Peshawar.

Describing another attempted murder, Sheeza shared,

When my friend tragically lost her life to gun violence, we reached out to her family to make arrangements for her remains. Unfortunately, they declined to accept the responsibility and cautioned us against bringing her body to their village, expressing concerns about potential ridicule that could tarnish our family’s honor. In no uncertain terms, they insisted that we should dispose of the deceased in the river, emphasizing their disassociation from her since the day she left their household.

Sheeza described that transgender people are not accepted by their families even on their death. This community keep fighting their battle from their birth till their deaths. This is such tragedy in transgender people’s lives that not only their only family members hate them, but many in the societies including their relatives and other cisgender people that are in their close circle do not accept their dead bodies and consider them a stigma and a sign of disrespect even after their deaths.

The study demonstrates that despite the protection provided in the legislation passed by national assembly (Transgender person welfare Act 2018) that aims to ensure the safety, security, and equality of transgender people in Pakistan, they still they are not accepted by their families from birth to death. Additionally, being the citizen of Pakistan, the constitution of the Islamic state of Pakistan provides protections of their fundamental rights, including security to every citizen (Article 9), avoids unwanted arrests and, if any, would be considered with prior notice (Article 10), inviolability of the dignity of man (Article 14), freedom of movement (Article 15), protection of property rights (Article 24), right to education (Article 25 A) and nondiscrimination in respect of access to public spaces (Article 26) (see the Constitution of Pakistan, 1972). But in the case of Khawaja Sara and Hijra, the federal and provincial governments are not able to provide the provision of fundamental rights, and in this way, they are routinely discriminated against, and frequently marginalized in their daily lives.

### Intersection of trans identity with police system

This theme describes how trans lives are understood with the intersection of the police system in Pakistan and how the ambiguity in police response affects transgender people in Pakistan. My argument seeks to examine the level of response and treatment that Khawaja Sara and Hijras (trans communities) get in legal procedural system in Pakistan, and how this treatment leads to discrimination and prejudice against transgender people in Pakistani societies.

Globally, transgender people are facing the highest rank of victimization. They are ranked at the top amongst other gender minority groups (Langenderfer-Magruder et al., 2016). Even in developed country like US, transgender people are 1.7% times more likely victimized than cisgender people (Peitzmeier et al., 2020). Similarly, in Canada, the statistics grow even worst than in the US. 59% of transgender people experience violence in their age 15 or above while the statics for cisgender people is 37% equal to the age of transgender people in Canada (Jaffray, 2020). In Australia, 17% of transgender people are victims of violence, including assault and rape (Boza & Nicholson Perry, 2014). Despite these alarming numbers still transgender people remain reluctant to visit police stations and to seek help from police against their victimization (Lee et al., 2023).

In Pakistan transgender communities too are not getting the ultimate respect from the police and they are therefore reluctant to visit police stations in Peshawar to report their First Information Reports (FIR’s). During a face-to-face interview, Jameela a senior guru in Peshawar has attested that police are always ridiculous toward them whenever they visit police station to lodge any complaints or to discuss any security issues. Despite the fact police are responsible to provide security and support to everyone irrespective of their creed, class, gender, and sexuality, this is not the case for transgender people in Pakistan as they are routinely ostracized and ridiculed in police stations. Jameela further added that,

When my house was burglarized in Peshawar city, I went to the local police station to file a First Information Report (FIR). However, despite seeking assistance, many individuals at the police station responded with laughter and did not take my case seriously. One police officer even insinuated that the robbery was a consequence of my involvement in certain activities, suggesting that I had taken advantage of people in dance parties, and now, in turn, faced a similar outcome. In essence, he expressed the sentiment, “da koyee hata wa awo pa koi olageeda” (pashtu quote) As you sow, so shall you reap (English translation).

Jameela highlights the negative behavior of police personal toward trans people and shows the non-serious attitude of police toward transgender people issues. This was also discussed in the study of Johnson et al., (2020), namely that Australian police are found nonresponsive, and they deal with cases related to LGBTIQ + communities differently. In this way, in many incidents, police in Australia remain a dominant social group against the LGBITQ + people that results in a negative outcome for them (Dwyer et al., 2017; Mennicke et al., 2018; Owen et al., 2018). This situation has developed a relationship of denial and rejection between police and LGBTIQ + communities in Australia (Miles-Johnson, 2016). In Pakistan, this was also discussed by Nisar (2016) in his PhD thesis,

Most of the Khawaja Sara with whom I talked during my research reported being arbitrarily stopped by police in urban public spaces as a significant aspect of their everyday life. These stops epitomize the hyper-visibility paradox for the Khawaja Sara. Many other research participants also noted there is often not even a made-up reason provided for the demand for money made by police officials at the stops. In fact, many times the stop is enacted just because the police officials find the Khawaja Sara easy victims to make some easy money due to their low socio-economic standing and social support.

Similarly, Nimo a young Cheela shared that people with a criminal background usually come to their houses and insist upon sex, and if this is resisted then they physical and mentally torture Cheela with guns and pistol.

Criminals forcefully enter our houses and subjecting us to abuse. Despite multiple visits to the police station to lodge FIR’s against them but no action has been taken so far by the police. “Mong hapal tool life ki insecure you zaka chez among jawond da masalo na dak dai. Pa di waja mong ziat tara hapala karma ki you” (Direct Pashto Quote). We are insecure in our whole life because we live in constant fear that disturb us mentally. We used to lock ourselves inside our rooms because we do not want to meet these criminals and afraid that they might harm us (English translation).

Nimo’s response is of great importance. She describes that transgender people are very innocent and powerless people. They can be easily harassed by cisgender people because many time their reports are not seriously taken by police. The offenders know that police and other law enforcing agencies would not put them in lockups, but they could easily resolve the matter with their influence or by offering them a handsome amount of money as bribery.

Additionally, Sweetie who is a Guru- (a senior member) discussed that,

The Khawaja Sara or Hijra communities encounter numerous challenges in their daily lives due to their transgender people identities. One prominent issue we face is the lack of adequate security, a matter of significant concern for these communities. Members of the Khawaja Sara or Hijra groups often find themselves vulnerable, both within their residences and during their participation in dance parties. Many times, we have been robbed and kidnapped while returning late night from our parties. Upon resisting many of our community members are physically assaulted or sexual abused, and in some cases, individuals have even been shot for resisting them. Regrettably, when such incidents are reported to the police, the response is often limited to filing a First Information Report (FIR), with little follow-up or further investigation. This lack of adequate security is exacerbated by societal misconceptions, as the broader community tends to associate these insecurities with the activities undertaken by the Khawaja Sara or Hijra communities, such as singing, dancing, and engaging in sex work. This unjust perception further contributes to the marginalization and vulnerability of these individuals, highlighting the urgent need for comprehensive measures to address the security concerns faced by the Khawaja Sara or Hijra communities.

Sweetie discusses the significant challenges faced by members of Khawaja Sara and Hijra communities in their daily lives in Peshawar. Firstly, the major concern highlighted is the pervasive lack of adequate security, posing a serious threat to the safety of these communities. Vulnerability is emphasized, both within their homes and during participation in activities such as dance parties. Instances of robbery, kidnapping, physical assault, and sexual abuse are distressingly common, especially when returning late at night from gatherings.

Secondly, the statement points out the alarming lack of responsiveness from law enforcement agencies when such incidents are reported. Despite filing FIRs, there is often minimal follow-up or thorough investigation. This inadequate security situation is compounded by societal misconceptions that unfairly link these insecurities to the activities of the Khawaja Sara or Hijra communities, such as singing, dancing, and engaging in sex work.

Last but not the least, the unjust societal perception exacerbates the marginalization and vulnerability of these individuals, emphasizing the pressing need for comprehensive measures to address the security concerns faced by the Khawaja Sara or Hijra communities. The statement calls for urgent attention and action to rectify these injustices and create a safer environment for these marginalized groups.

Kanwal asserted that,

We get no support from police and other district administration. Even in some districts of Khyber Pukhtunkhawa people like us, are forcefully kicked out from their Deras or Houses after imposing section-144^5^ from police that restrict our movements, and we are no longer to perform our events or activities. Similarly, if police find any transgender people in criminal activities, they beat them brutally and also lock them in male prisons. In Peshawar no separate prison or lockups are available for transgender people and when we are kept in male lockups, we are easily sexually harassed by other prisoner in lockup.

Kanwal discusses the profound lack of support and systemic discrimination faced by the transgender community from police and district administration, particularly in certain districts of Khyber Pakhtunkhwa. She describes different circumstances of transgender people who are are forcibly expelled from their guru-cheela houses or their movements and gathering places are restricted when the administration imposes section 144 i that severely restricts the freedom of movement of transgender people and prevents them from organizing their events and activities.

The quote also sheds light on the harsh treatment transgender individuals receive from law enforcement agencies. In cases where a transgender person is suspected or involved in criminal activities, the police resort to brutal beatings and detaining them in male prisons. The absence of separate facilities for transgender individuals in places like Peshawar exposes them to further vulnerability, as they are housed in male lockups where they become easy targets for sexual harassment from other inmates.

Nirma, a young Khawaja Sara Cheela who describes the story of her friend in a face-to-face interview conducted at her place called Dera^6^,

My friend was in a long-term relationship with her partner for several years. Tragically, she was fatally shot along with her friend while returning from a night party, the perpetrator being her own partner in Peshawar. Although we promptly filed a case with the local police station, no significant progress has been made to date. Unfortunately, this is not an isolated incident, as numerous similar cases have been previously reported in pursuit of justice through the police and judiciary systems, yet none have seen a resolution with fair and just outcomes.

Nirma illustrated the story of a transgender friend when she was assassinated and shot dead by her intimate partner and how her case was taken by the police. This shows the lack of interest and level of seriousness to deal with the cases of transgender communities in Pakistan. The local social, cultural, and religious prejudice on the part of police personal continuously fails to provide security and justice to transgender people in Peshawar. She further added that even going to the police station, they are regularly harassed, and police make jokes and make fun of then. During the procedural process many of the responsible authorities laugh at transgender people.

The above extensive discussion explores the intersection of transgender person lives in Pakistan with the police system, shedding light on the pervasive discrimination and challenges faced by Khawaja Sara and Hijra communities. The argument delves into the inadequate response and treatment within the criminal justice system, leading to heightened discrimination and prejudice against transgender people in Pakistani society.

### Intersection of trans identity with Judiciary and courts

This section investigates transgender people’s experience within the context of the criminal justice system in Pakistan whilst using an intersectional lens to describe how transgender people known as Khawaja Sara and Hijra are marginalized by the legal actors including advocates and judiciary courts. The intersectional approach provides a glimpse of multiplicity in the oppression and discrimination of transgender people lives in Peshawar city. The data in this section attest that transgender people would not be able to get fairness and justice until the legal actors or the justice system treat them with dignity. So far, transgender people give the impression that they are not equally treated and even their cases are not taken seriously in the judiciary courts. This highlights the nuance in the criminal justice system in Pakistan against the legal experience of transgender communities.

Research scholarship does not favor the relationship between transgender people and legal actors (Stotzer et al., 2014). A study by Buist and Stone (2014) demonstrates that transgender people are treated unfairly in comparison to cisgender people. My findings also testify that Khawaja Sara and Hijra as transgender people are regularly harassed in their daily routine life, and they do not get the proper attention in police stations and judiciary courts. Research by Ezie (2023) explains that transgender people are presumed as sex workers, and they are arrested without any cause and charge. Avalos uses the term ‘axes of oppression’ because transgender people are subject to frequent oppression and violence due to their marginalized identities.

Transgender people in Pakistan have received recognition through the Transgender Persons (Protection of rights) Act-2018 that helps them to be safe, provides medical and educational facilities, and also allows government to establish centers to provide them psychotherapy. This was made possible when the wave of trans activism started in 2009 when some transgender people were arrested from a dance function in Pakistan. In reaction to this arrest and later on reported violent treatment in police custody, many transgender people attacked the respective police station In these circumstances, a jurist advocate “Aslam Khaki” filed a petition in the supreme court of Pakistan and echoed that transgender people should be given their basic human rights in light of the Constitution of Pakistan. The Supreme Court (SC) gave their famous verdicts like right to education, right to health and right to vote to transgender people in Pakistan in the year between 2009-2012. The most important development after this case was the legislation of “transgender person identity” to legally acknowledge the distinctive identity of transgender people in Pakistan. In its last verdict, the SC announced that the “rights including right to life and dignity of the (Khawaja Sara community) are equally protected (by the constitution). Thus, no discrimination, for any reason, is permissible to the extent that their rights and obligations are concerned.” Where the supreme court of Pakistan verdict had its constraint particularly in its verbalization of the “transgender person identity” as being biologically based, it was generally recognized as an optimistic movement toward the social inclusion of the Khawaja Sara and Hijra in Pakistan.

Despite the recent legislation, transgender people in Peshawar have still many problems in judiciary and public courts that put questions on the criminal justice system in Pakistan. In many judicial proceedings, no specialized courts are available to deal or pursue transgender people’s cases. Additionally, transgender people face many financial issues when hiring advocates as they are unable to pay huge amount of money. In this way, transgender people’s cases are either withdrawn as no one is willing to pursue them or either their families do settlement with the offender as blood money and the (offender) easily then gets bail from the court.

A local media agency (BBC news, 2019)^7^ in Peshawar has reported different cases where transgender people are killed either by their friends, intimate partners or family members, and the cases did not get any attention or further proceedings.

Nazo, a transgender person in Peshawar, was killed by two friends in July last year. They hacked her body into pieces, stuffed it in plastic bags and were carrying it for disposal when police caught them. Nazo’s family considered it below their dignity to accept her body, so it was buried in a police graveyard in Peshawar. But they did feel they owned Nazo when they were offered blood money by the killers in return for filing a pardon in court. The two men were acquitted on the basis of that pardon in months.

In a similar transgender person case,

A trans woman from Mardan^8^ was allegedly killed by her family. Though pictures of her dead body were circulated by rights activists on social media, no-one filed a murder case with the police, nor did the police bother to act on the tip.

In another case,

Maya a transgender person was killed near Peshawar. Police did not include the honour killing clause in her case that produce many chances to Maya’s mother to pardon the killer and they literally did that where the offender got bail easily at the end of the case. Maya was killed by her father in the name of honour, but her murder was not included in terms of honour killing and her father easily got bail from the court.

This was attested by Shanza in a face-to-face interview with the author,

When we visit the district courts, everyone there passes comments on us. “Mong che halak ovene no bus handa mong poory shuru ki, pa di waje mong aksar hapal korona ki you” (Direct Pashto quote) When people see us, they always laugh at us therefore, we most of time pass our time inside our house and not go outside (English translation). Last, our families do not accept us, so we became isolated and more vulnerable to all these difficulties that we face in our daily life,

Shanza’s response illustrates that factors like stigma, ridiculing, discrimination, and oppression are intersected within the procedural process in the criminal justice system in the context of transgender people. She further demonstrates that many people in the judiciary and court rooms make fun of them, and they pass comments that humiliate them. This is because of the cisgenderist nature of law enforcement officials and officers and staff. Wolff and Cokely (2007) report that due to potential transphobia, many gender minorities are embarrassed on the hands of prosecutors, and other law enforcement offices who they interact in the proceedings of their cases. In this way, it hinders the willingness of victims, resulting in their marginalization and oppression in their cases (Mallory et al., 2015).

Similarly, Nimo discussed that,

We are granted right to vote, right to equal education and better facilities of health in the areas where we are living but no institute has complied with the verdict of Supreme Court. This shows the lack of response from the devolved departments and District and provincial courts who failed to provide us the facilities that are already lauded by the top ranked court in Pakistan.

Nimo has described how she is discriminated in her life and how the responsible authorities have shown a lack of interest in the provision of equality in education and good facilities of health and employment to transgender communities in Pakistan. But despite this, transgender communities are not getting their due justice in Pakistan.

Jameela, who is guru, shared that she has both good and bad experiences with the criminal justice system in Pakistan. She discussed that,

Criminal justice in Pakistan is failed to provide us protection and security to our lives and properties. My cheela was killed and I filed her FIR, I did press conference against the police department, but they made pressure on me. I got many threats from the police to withdraw the case, but I did not do that. Police arrested the offender and within next few weeks district court granted bail to him, and he is living freely now.

Jameela illustrated how the police department and court system in Pakistan is inadequate in dealing transgender issues. This shows that transgender people are made more precarious and soft targets for the offenders because they have the support of police, and also the courts provides them easy bails within weeks. The offenders then become more threatening for transgender people in Peshawar. The statistics of HRCP shows that more than 55 Khawaja Sara and Hijra have been killed within the last five years in Peshawar, and none of the offenders were punished, but instead were out of prison on bails and living their life freely.

Sweetie shared that she is the chairperson of an organization that is working for the advocacy and mobilization of transgender communities in Peshawar. During her interview, she discussed that she filed a petition in Peshawar Hight Court (PHC) and demanded for the allocation of a special quota for transgender people in the electoral system so that they may be able to represent their communities in the national and provincial assemblies. But despite this petition it is till this day pending with the Peshawar Hight Court,showing the negligence of transgender people’s demands and issues in the justice system. Sweetie stated,

I am the senior most transgender person in Peshawar and I am working for the better of this community. I am running a local organisation that mobilise them and also works for the solution of their day-to-day problems. As we have not got the right to vote in the polling process, I filed a petition to for allocation of special quota for us in both the national and provincial assemblies.

Similarly, Saima another senior member of transgender communities in Peshawar has discussed that,

We submitted a writ petition to Peshawar Hight Court in 2018 requesting to direct the Election Commission of Pakistan to make the political process easier friendly for transgender people and to ensure their both as voter and also as a returned candidate in the elections. But despite of all these efforts the decision did not turn in our favour but was kept pending and went on without specific direction for many months.

Kinza further describes how a person was granted bail by Peshawar High Court after killing a friend of hers, and the other person was injured even after the report of injured eyewitness.

My friends were returning back from their function and all at the sudden this person opened fired on them killing my friend and the other got bullets on her hands, feet and back regions. Being the injured of the incident and the prime eye witness the police arrested the petitioner but the PHC granted bail to the offender contended that the incident happened at night-time where the attacker was not visible to the victims. The court let the offender free on bail with the remarks that the investigation officer had poorly conducted the investigation and falsely implicated the petitioner.

Participant responses highlight that transgender people are not willing to visit police station to register their FIR’s and district court to report their incidents even if they have faced robbery, kidnapping, harassment and even killing. They are reluctant to visit a police station because they have no trust on these institutions. Using the intersectional theoretical framework, this study explains the precarious identities of Khawaja Sara and Hijra individuals within the intersection of police stations and district court responses that exacerbate the challenges they face in their daily lives. The findings reveal that transgender people are denied robust familial connections and personal relationships, such as those with parents and siblings, hindering their ability to navigate legal proceedings or receive emotional, social, and financial support. This absence of support further intensifies the vulnerabilities they experience in their lives.

## Discussion

Intersectionality is considered very important because any analysis that does not take intersectionality into account cannot sufficiently address how identities are subordinated (Crenshaw, 1990). The data justifies that discrimination against Khawaja Sara and Hijra is not solely anchored in their gender identity, but is also exacerbated by socio-cultural norms and religious beliefs. This multifaceted discrimination manifests in biased treatment within their communities and the criminal justice system, adversely affecting their quality of life, impeding their access to justice, and perpetuating systemic injustices.

Khawaja Sara and Hijra are stigmatized within their families, experience negative responses that affect their access to public services. This, in turn, impacts their physical health, lifestyle, education, and personal security. Discrimination extends beyond the family to the broader community, where transgender individuals are subjected to ridicule and disrespect in various aspects of life, making their daily existence challenging.

Comparatively, studies from the UK and Australia reveal that LGBTIQ + individuals intentionally avoid institutions like the police and judiciary due to fear of oppression and stigmatization. The studies of Leonard et al. (2008), Miles-Johnson (2016), and Owen et al. (2018), described that LGBTIQ + people in UK and Australia intentionally avoid visiting or contacting institutions like police and judiciary because of their fear of oppression and stigmatization. Extant research demonstrates that trans people face poor treatment when they seek justice compared to their cisgender counterparts (Buist & Stone, 2014; Stotzer, 2014). Similarly, the trend exists in countries like the United States and Canada, where high rates of violence against transgender people coexist with a reluctance to seek help from law enforcement, highlighting systemic mistrust and discrimination.

A study conducted by Van Hout et al. (2020) in the United States has underscored the challenges faced by transgender individuals in correctional facilities, and has advocated for the provision of enhanced prison facilities to ensure better management for this demographic. Similarly, the review by Brömdal et al. (2019), spanning both the United States and Australia, has illuminated the arduous experiences of transgender prisoners in comparison to their non-transgender counterparts within correctional settings. The “otherness” of transgender individuals becomes a weapon wielded against them, not only by fellow inmates through intimidation and violence, including sexual assault, but also by prison officers who contribute to this mistreatment through neglect and ignorance.

In Pakistan, transgender communities face a lack of respect from the police, deterring them from reporting incidents. Personal accounts, such as Jameela’s experience of being ridiculed while filing an FIR, expose discriminatory attitudes within the police force. The intersectionality of transgender identity, socio-economic status, and societal prejudices is evident in their interactions with the police, where demands for money further illustrate their marginalized status in Peshawar. Applying Crenshaw’s theory of intersectionality (1990) to the criminal justice system unveils layered challenges faced by transgender individuals in Peshawar Pakistan. Beyond issues related to gender identity, they contend with intersecting factors such as socio-cultural and religious norms. This intersectionality emphasizes the need to consider these multiple axes of identity to understand the unique vulnerabilities faced by transgender people in the criminal justice system.

Within the transgender community, varied experiences exist, acknowledging that individuals face discrimination to different degrees based on factors like socio-cultural norms. Engagement with the police reveals that intersecting identities produce new challenges, hindering their pursuit of justice in Peshawar. Despite support from like-minded individuals, fear for their lives and the potential for increased violence post-release deters transgender individuals from pursuing their cases. Understanding the experiences through the lens of intersectionality underscores the essential need for comprehensive policies and interventions. Such an approach recognizes the interconnected nature of social categories and aims to dismantle the various layers of discrimination transgender individuals encounter. By fostering a more equitable and just criminal justice system in Pakistan, these policies can contribute to a safer and more inclusive society for transgender communities.

### Limitation of the study

In exploring the intersection of transgender people and the criminal justice system in Pakistan, this study has certain limitations. Firstly, the research was conducted with a small sample size, selecting 10 members from Khawaja Sara and Hijra communities in Peshawar because accessing transgender people and to select a comprehensive sample was a challenge in Peshawar due to the reluctance of participants to share their personal experiences. The study also encountered challenges in obtaining the official records or documentation related to transgender individuals within the criminal justice system, potentially limiting the depth of analysis. Furthermore, the dynamic nature of legal and policy frameworks impacted the generalizability of the findings over time. Despite these limitations, the study endeavored to shed light on an important but understudied area, acknowledging the complexities inherent in researching the experiences of transgender individuals within the criminal justice system in the context of Pakistan.

## Conclusion

Numerous research studies have explored the challenges faced by transgender communities in South Asian countries such as Pakistan, India, and Bangladesh, particularly in the realms of health, education, civic engagement, and economic well-being. However, a critical gap exists as no specific research have investigated the intersection of transgender people and the criminal justice system in Pakistan, with a focus on Pashtun culture in Peshawar city.

This study not only discussed the persistent issues, such as frequent harassment, physical and sexual abuse, discrimination, and mistreatment faced by transgender individuals in their broader communities, but also explored how trans people are subjected to the inadequacies of the criminal justice system in addressing trans-specific issues in Peshawar. It is revealed that transgender individuals are often regarded as bearers of social stigma, perceived as threats to family honor, which results in their expulsion from their parental homes. Subsequently, their lives become fraught with challenges, exposing them to various forms of structural, social, and institutional violence in their daily existence. This heightened vulnerability places transgender communities at an increased risk of involvement in the legal system and encountering negative treatment from legal entities.

Notably, transgender individuals exhibit hesitancy in approaching police stations or judicial courts to seek assistance or report incidents, given the fragile relationships they often maintain with family members. The societal isolation resulting from their being trans makes them insecure, especially when navigating public spaces without adequate support. Instances of harassment and abuse, including those perpetrated by individuals within the police and other law enforcement agencies, contribute to a cycle wherein the response to trans people become notorious. Offenders may receive lenient charges or obtain bail even for severe criminal acts such as harassment, robbery, abuse, or homicide. Many transgender individuals refrain from engaging with law enforcement agencies due to a lack of trust in the criminal justice system. Their reluctance is further fueled by the fear of exposing incidents that could tarnish their family’s honor, leading them to keep such matters discreet. Moreover, the transphobic and cisgenderist environment in Peshawar inhibits the visibility of their issues and hampers their quest for justice in their daily lives.

## Notes

1 Children Act (2002) is a proactive measure to curb acts of violence against women and children, Bangladesh enacted the Prevention of Oppression against Women and Children Act.

2 Aurat Foundation is a non-profit organisation working in Pakistan for the empowerment of vulnerable communities and those who are in need of help and support.

3 Pakistan inherited the criminal justice system from 90 years of British rule, is given in the Criminal Procedure Code (CrPC), 1898 which provides for case registration and investigation by the police and the trial in a criminal court. Its basic objective is to provide protection to life and property of citizens and to ensure order in society. It has three main phases: investigation by police, trial by Courts, and execution by jail authorities. It stands upon three pillars: investigation, prosecution, and trial.

4 Cheela is a young Khawaja Sara or Hijra who newly entered the guru-cheela houses in Peshawar.

5 Section 144 of the Code of Criminal Procedure (CrPC) empowers district administration to issue orders in public interest that may place a ban on an activity for a specific period of time.

6 Dera is house or place of living of Khawaja Sara and Hijra.

7 https://www.bbc.com/news/world-asia-50486439 accessed on 4^th^ April, 2023.

8 Mardan is one of the districts in Peshawar.

## References

[CIT0001] Anuvinda, P., & Siva, T. (2016). No country for transgenders? *Economic and Political Weekly*, *51*(37), 19–22.

[CIT0002] Avalos, S. (2022). The trans experience with the criminal legal system. *Crime & Delinquency*. 10.1177/00111287221134914

[CIT0003] Awan, M. A. (2019). Transgender people and human rights issues in Pakistan [Doctoral dissertation]. Johann Wolfgang Goethe-Universität Frankfurt am Main.

[CIT0004] Badgett, M. V., Lau, H., Sears, B., & Ho, D. (2007). *Bias in the workplace: Consistent evidence of sexual orientation and gender identity discrimination*. UCLA: The Williams Institute.

[CIT0005] Benson, K. (2020). What’s in a pronoun? The ungovernability and misgendering of trans Native kids in juvenile justice in Washington State. *Journal of Homosexuality*, *67*(12), 1691–1712. 10.1080/00918369.2019.1613854 31116672

[CIT0006] Boza, C., & Nicholson Perry, K. (2014). Gender-related victimization perceived social support, and predictors of depression among transgender Australians. *International Journal of Transgenderism*, *15*(1), 35–52. 10.1080/15532739.2014.890558

[CIT0007] Braun, V., & Clarke, V. (2006). Using thematic analysis in psychology. *Qualitative Research in Psychology*, *3*(2), 77–101. 10.1191/1478088706qp063oa

[CIT0008] Brömdal, A., Mullens, A. B., Phillips, T. M., & Gow, J. (2019). Experiences of transgender prisoners and their knowledge, attitudes, and practices regarding sexual behaviors and HIV/STIs: A systematic review. *The International Journal of Transgenderism*, *20*(1), 4–20. 10.1080/15532739.2018.1538838 32999591 PMC6830990

[CIT0009] Buist, C. L., & Stone, C. (2014). Transgender victims and offenders: Failures of the United States criminal justice system and the necessity of queer criminology. *Critical Criminology*, *22*(1), 35–47. 10.1007/s10612-013-9224-1

[CIT0010] Crenshaw, K. (1989). Demarginalizing the intersection of race and sex; a Black feminist critique of discrimination doctrine, feminist theory and antiracist politics. *University of Chicago Legal Forum*, *1989*(1), 139–167.

[CIT0012] de Lind van Wijngaarden, J. W., Schunter, B. T., & Iqbal, Q. (2013). Sexual abuse, social stigma and HIV vulnerability among young feminised men in Lahore and Karachi, Pakistan. *Culture, Health & Sexuality*, *15*(1), 73–84. 10.1080/13691058.2012.743186 23157327

[CIT0013] Dwyer, A. E., Ball, M. J., Bond, C., Lee, M., & Crofts, T. (2017). Reporting Victimization to LGBTI (Lesbian, Gay, Bisexual, Transgender, Intersex) Police Liaison Services: A Mixed Methods Study across Two Australian States. Report to the Criminology Research Advisory Council Grant: CRG 31/11-12. Canberra: Criminology Research Advisory Council.

[CIT0014] Ellis, S. J., Bailey, L., & McNeil, J. (2016). Transphobic victimisation and perceptions of future risk: A large-scale study of the experiences of trans people in the UK. *Psychology & Sexuality*, *7*(3), 211–224. 10.1080/19419899.2016.1181669

[CIT0015] Ezie, C. (2023). Dismantling the discrimination to incarceration pipeline for trans people of color. *University of St. Thomas Law Journal*, *19*.

[CIT0017] Islam, S. (2019). A theoretical analysis of the legal status of transgender: Bangladesh perspective. *International Journal of Research and Innovation in Social Science*, *3*(3), 117–119.

[CIT1002] Jaffray, B. (2020). Experiences of violent victimization and unwanted sexual behaviours among gay, lesbian, bisexual and other sexual minority people, and the transgender population, in Canada, 2018. Juristat: Canadian Centre for Justice Statistics, 1-27.

[CIT0018] Jami, H. (2005). Condition and status of Hijras (transgender, transvestites etc) in Pakistan [Paper presentation]. Sexualities, Genders and Rights in Asia. 1st International Conference of Asian Queer Studies, 2006.

[CIT1004] Johnson, C., Girgis, A., Paul, C., Currow, D., Adams, J., & Aranda, S. (2020). Australian palliative care providers’ perceptions and experiences of the barriers and facilitators to palliative care provision. *Support Care Cancer, 19*, 343–351. 10.1007/s00520-010-0822-0 20157747

[CIT0019] Kakakhel, S. F. (2022, September 18). More violence. *The News Sunday*. https://www.thenews.com.pk/tns/detail/992139

[CIT1001] Khan, A. A., Rehan, N., Qayyum, K., & Khan, A. (2008). Correlates and prevalence of HI and sexually transmitted infections among Hijras (male transgenders) in Pakistan. *International Journal of STD & AIDS, 19*(12), 817–820. 10.1258/ijsa.2008.008135 19050211

[CIT0020] Khan, A. U. (2016). Gendered justice: Constitutions, trans-genders, and equality. *LUMS Law Journal*, *3*, 69.

[CIT0021] Khan, A. (2022, March 17). Transgender shot dead over local dispute in Mardan. *The Express Tribune*. https://tribune.com.pk/story/2348441/1?fbclid=IwAR2HhQ.

[CIT0022] Kilbride, E. (2015). The forgotten: Pakistan’s transgender population, and the Islamic State. E-International Relations. https://www.eir.info.com.pk

[CIT0023] King, S. (2019). The criminal justice system’s mistreatment of transgender individuals: A call for policy reform to assist a marginalized prisoner community. *Inquiries Journal*, *11*(1).

[CIT0024] Langenderfer-Magruder, L., Whitfield, D. L., Walls, N. E., Kattari, S. K., & Ramos, D. (2016). Experiences of intimate partner violence and subsequent police reporting among lesbian, gay, bisexual, transgender, and queer adults in Colorado: Comparing rates of cisgender and transgender victimization. *Journal of Interpersonal Violence*, *31*(5), 855–871. 10.1177/0886260514556767 25392392

[CIT1003] Lee, Y. J., & Santiago, L. (2023). Race, class, and gender identity: implications for transgender people’s police help seeking. *Police Practice and Research, 24*(1), 17–31.

[CIT1005] Leonard, W., Pitts, M., Mitchell, A., & Patel, S. (2008). *Coming forward: The underreporting of heterosexist violence and same sex partner abuse in Victoria*.

[CIT0028] M’baye, B. (2013). The origins of Senegalese homophobia: Discourses on homosexuals and transgender people in colonial and postcolonial Senegal. *African Studies Review*, *56*(2), 109–128. 10.1017/asr.2013.44

[CIT0029] Majeedullah, A. (2016). *Living on the periphery: The Khawaja Siras of Pakistan. (No. IDS Evidence Report; 165)*. IDS.

[CIT0030] Mallory, C., Hasenbush, A., & Sears, B. (2015). *Discrimination and harassment by law enforcement officers in the LGBT community*. UCLA: The Williams Institute.

[CIT0031] Mennicke, A., Gromer, J., Oehme, K., & MacConnie, L. (2018). Workplace experiences of gay and lesbian criminal justice officers in the United States: A qualitative investigation of officers attending a LGBT law enforcement conference. *Policing and Society*, *28*(6), 712–729. 10.1080/10439463.2016.1238918

[CIT0032] Miles-Johnson, T. (2016). Perceptions of group value: How Australian transgender people view policing. *Policing and Society*, *26*(6), 605–626. 10.1080/10439463.2014.996563

[CIT0033] Mitra, P. (2018). Human rights violation of transgender people: A critical analysis on Bangladesh perspective. *Kathmandu School of Law Review*, *6*, 165–175. 10.46985/jms.v6i2.212

[CIT0034] Mogul-Adlin, H. (2015). Unanticipated: Healthcare experiences of gender nonbinary patients and suggestions for inclusive care [Doctoral dissertation]. Yale University.

[CIT0035] Nanda, S. (1986). The Hijras of India: Cultural and individual dimensions of an institutionalized third gender role. *Journal of Homosexuality*, *11*(3-4), 35–54. 10.1300/J082v11n03_03 4093603

[CIT0036] Nanda, S. (1990). *Neither man nor woman: The hijras of India*. Wadsworth.

[CIT0037] Nisar, M. A. (2016). Managing the margins: Intersections of the state and the Khawaja Sira in Lahore, Pakistan [Doctoral dissertation]. Arizona State University.

[CIT0038] Owen, S. S., Burke, T. W., Few-Demo, A. L., & Natwick, J. (2018). Perceptions of the police by LGBT communities. *American Journal of Criminal Justice*, *43*(3), 668–693. 10.1007/s12103-017-9420-8

[CIT0039] Peitzmeier, S. M., Malik, M., Kattari, S. K., Marrow, E., Stephenson, R., Agénor, M., & Reisner, S. L. (2020). Intimate partner violence in transgender populations: Systematic review and meta-analysis of prevalence and correlates. *American Journal of Public Health*, *110*(9), e1–e14. 10.2105/AJPH.2020.305774 PMC7427218 32673114

[CIT0040] Reddy, G. (2005). Geographies of contagion: Hijras, Kothis, and the politics of sexual marginality in Hyderabad. *Anthropology & Medicine*, *12*(3), 255–270. 10.1080/13648470500291410 26873670

[CIT0041] Saddique, K. A. M. R. A. N., Mirbehar, S., Batool, H., Ahmad, I., & Gang, C. (2017). Transgender issues in Pakistani community. *European Academic Research*, *4*(10), 9048–9057.

[CIT0042] Stotzer, R. L. (2009). Violence against transgender people: A review of United States data. *Aggression and Violent Behavior*, *14*(3), 170–179. 10.1016/j.avb.2009.01.006

[CIT0043] Stotzer, R. L., Herman, J. L., & Hasenbush, A. (2014). *Transgender parenting: A review of existing research*. The Williams Institute.

[CIT1006] Stotzer, R. L. (2014). Law enforcement and criminal justice personnel interactions with transgender people in the United States: A literature review. *Aggression and Violent Behavior, 19*(3), 263–277.

[CIT0044] Taşcıoğlu, E. (2023). Circuits of Law: Everyday criminalisation of transgender embodiment in istanbul. In *Criminal legalities and minorities in the global south: Rights and resistance in a decolonial world* (pp. 231–251). Springer International Publishing.

[CIT0045] Van Hout, M. C., Kewley, S., & Hillis, A. (2020). Contemporary transgender health experience and health situation in prisons: A scoping review of extant published literature (2000–2019). *International Journal of Transgender Health*, *21*(3), 258–306. 10.1080/26895269.2020.1772937 34993510 PMC8726645

[CIT0046] Walters, M. A., Paterson, J., Brown, R., & McDonnell, L. (2020). Hate crimes against trans people: Assessing emotions, behaviors, and attitudes toward criminal justice agencies. *Journal of Interpersonal Violence*, *35*(21-22), 4583–4613. 10.1177/0886260517715026 29294810

[CIT0047] Wolff, K. B., & Cokely, C. L. (2007). “To protect and to serve?”: An exploration of police conduct in relation to the gay, lesbian, bisexual, and transgender community. *Sexuality and Culture*, *11*(2), 1–23. 10.1007/s12119-007-9000-z

